# A high spatial resolution dataset of China’s biomass resource potential

**DOI:** 10.1038/s41597-023-02227-7

**Published:** 2023-06-15

**Authors:** Rui Wang, Wenjia Cai, Le Yu, Wei Li, Lei Zhu, Bowen Cao, Jin Li, Jianxiang Shen, Shihui Zhang, Yaoyu Nie, Can Wang

**Affiliations:** 1 Department of Earth System Science, Institute for Global Change Studies, Ministry of Education Ecological Field Station for East Asian Migratory Birds, Tsinghua University, Beijing, 100084 China; 2 State Key Joint Laboratory of Environment Simulation and Pollution Control (SKLESPC), School of Environment, Tsinghua University, Beijing, 100084 China; 3 PBC School of Finance, Tsinghua University, Beijing, 100084 China; 4 Beijing E-Hualu Information Technology Co., Ltd, Beijing, 100043 China

**Keywords:** Climate-change mitigation, Climate and Earth system modelling

## Abstract

Assessing biomass resource potential is essential for China’s ambitious goals of carbon neutrality, rural revitalization, and poverty eradication. To fill the data gap of high spatial resolution biomass resources in China, this study estimates the biomass resource potential for all types of lignocellulosic biomass feedstock at 1 km resolution in 2018, including 9 types of agricultural residues, 11 types of forestry residues, and 5 types of energy crops. By combining the statistical accounting method and the GIS-based method, this study develops a transparent and comprehensive assessment framework, which is in accordance with the principle of food security, forest land and pasture protection, and biodiversity protection. In the end, we organize and store the data in different formats (GeoTIFF, NetCDF, and Excel) for GIS users, integrated modelers, and policymakers. The reliability of this high spatial resolution dataset has been proved by comparing the aggregated data at the subnational and national levels with the existing literature. This dataset has numerous potential uses and is a crucial input to many bioenergy-related studies.

## Background & Summary

Utilizing biomass resources effectively is crucial for limiting climate change, as bioenergy is an important net-zero renewable energy. When coupled with carbon capture and storage, it can also remove the carbon in the atmosphere^1,2^. Besides, making full use of agricultural and forestry residues can avoid air pollution caused by open biomass burning^3,4^, and also encourage the growth of the recycling economy^5^. Additionally, planting perennial grass on degraded land could improve soil condition^6,7^ and spur rural development^8^. China has established ambitious goals of carbon neutrality, rural revitalization, and poverty eradication in recent years. As the third large country in the world in terms of land area, with 1.34 million km^2^ of arable land and a 24% forest coverage rate, China has a vast biomass resource potential that has yet to be tapped. Therefore, it is imperative to evaluate how many biomass resources are available in China.

However, current studies may provide limited insights into policy implications and bioenergy deployment for the following three reasons. First, current studies only focus on one or a few types of biomass resources, such as agricultural residues^9–13^, forestry residues^14,15^, or energy crops^16–19^, or assess biomass resource potential for a specific region^20,21^, which prevents us from having a comprehensive understanding of the potential of biomass resources in China. Second, few studies provide high-resolution maps for each type of agricultural residue (e.g., rice straw map, wheat straw map, cotton straw map, etc.), forestry residues (e.g., logging residues from shrub land, sparse land, economic forest, etc.), and energy crops (e.g., switchgrass map on abandoned cropland, miscanthus map on marginal land). In addition, they pay little attention to the policy constraints, such as ensuring food security, forest land and pasture protection, and biodiversity protection. This may lead to an overestimation of the potential of biomass resources, prevent us from identifying the predominant types of biomass resources for each province, and cannot provide targeted instruction for each land on biomass resource development. Last and most importantly, to the best of our knowledge, there is no publicly available biomass resources dataset with high spatial resolutions^22–24^, which is crucial for many Integrated Assessment Models (IAMs) and bioenergy deployment studies. Besides, this data gap also prevents the direct comparison and rectification of various biomass resource assessments.

Therefore, our study aims to publish a high spatial resolution dataset of biomass resources for China. This dataset fully considers the availability of China’s land use and multiple types of feedstock types, including 9 types of agricultural residues, 11 types of forestry residues, and 5 types of energy crops. By integrating the statistical accounting method and GIS-based method, this study develops a clear and transparent framework for high spatial resolution assessment of biomass resource potential, which can be applied to different countries or regions. In the end, we organize the data in different formats (GeoTIFF, NetCDF, and Excel) for other discipline researchers and policymakers.

This dataset can be used from three perspectives. First, this dataset shows the spatial distribution of biomass resources, which can indicate where to collect agricultural and forestry residues for bioenergy production (e.g. bio-diesel, bio-ethanol, bio-electricity, bio-gas, bio-jet fuel, etc.) and where to plant energy crops in the future. Second, this dataset can be used as the input dataset for many IAMs to evaluate the influence of large-scale bioenergy development on the climate, economy, and energy system at the sub-national level^25–27^. Third, it can serve as the foundational dataset for bioenergy deployment studies, including bio-refinery siting^28^, bioenergy supply chain design^29^, and bioenergy industry layout^30^, etc.

## Methods

**Fig. 1:**
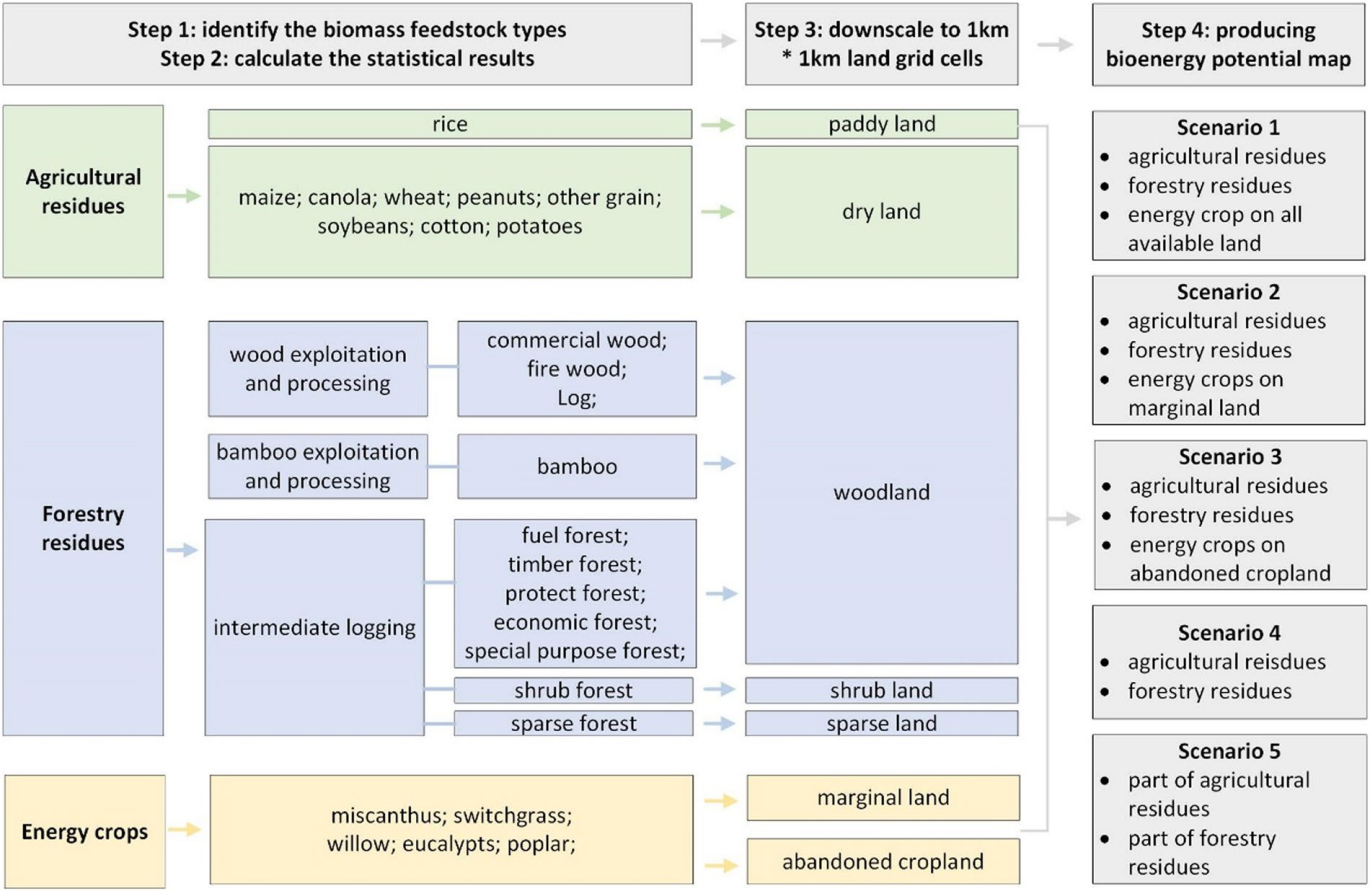
Overview of biomass resource potential assessment procedures.

The biomass resource dataset consists of three types, including agricultural residues, forestry residues, and energy crops as shown in Fig. 1. Agricultural residues are sourced from 9 types of agricultural products (Section 2). Forestry residues consist of the residues from wood exploitation and processes, bamboo exploitation and processes, and forest logging processes (Section 3). Energy crops include 2 types of herbaceous crops and 3 types of woody crops. These energy crops are planted on marginal land, which cannot encroach on arable land, major forest land, and pasture, cannot have a conflict with natural reserves, and should not be planted in areas prone to soil erosion (Section 4). Based on the above context, we collect the statistical data, a number of the province- and feedstock-specific parameters, and a series of land-related datasets. By combining GIS and statistical methods, we obtain various types of high spatial resolution maps for biomass resources. In the end, considering different utilization degrees, we design five scenarios for data aggregation. The utilization degree of biomass resources decreases from scenario 1 to scenario 5 (Section 5).

### Basic datasets preparation

#### Spatial dataset collection

To assess biomass resources at a high spatial resolution, we collect five land-related datasets, including land use maps, natural reserves, slope data, net primary production dataset, and energy crops yield map.

1. land use map
  This study collects the land use map in 1980 and 2018 from the Resource and Environment Science and Data Centre of the Chinese Academy of Science (https://www.resdc.cn/DOI/DOI.aspx?DOIID=54). The land use map includes 6 primary classes and 25 subclasses. First-class land consists of agricultural land, forestry land, grassland, water area, residential area, and unused land. The agricultural land includes paddy land and dry land. The forest land is categorized into woodland, shrub land, sparse land, and other forest lands by canopy density. The grassland is divided into high, medium, and low coverage. Unused land consists of saline soil land, bare soil land, etc. In the following evaluation procedure, the land use map will be utilized for marginal land identification, spatial allocation of statistical results, and the forest logging residues assessment of sparse land and shrub land.
2. natural reserve
  The natural reserve dataset also sources from RESCD (https://www.resdc.cn/data.aspx?DATAID=272), which includes wildlife sanctuaries, forest ecological reserves, geological heritage reserves, paleontological remains reserves, inland wetland reserves, and marine coastal reserves. The total area is 1.007 million km^2^. As nature reserves have important ecological values, China’s administrative regulations prohibit a range of activities in these areas, such as logging, land clearing, business, and so on. Therefore, these areas cannot be used for energy crop plantation.
3. digital elevation model data
  The production of digital elevation model data is based on the Shuttle Radar Topography Mission data from the U.S. Space Shuttle Endeavour (https://www.resdc.cn/data.aspx?DATAID=123). It can be used to calculate the slope for each grid cell with the slope tool in ArcMap. As China’s soil and water conservation law prohibits the cultivation of crops on a steep slope above 25 degrees, and many studies also show that severe soil erosion will occur in areas with a slope greater than 25 degrees^31–33^, this study excludes these areas for energy crop plantation.
4. net primary production dataset

In the process of plant growth, part of the energy fixed by the plant will be consumed by self-respiration. The remaining energy used for growth and reproduction is called net primary production (NPP)^34,35^. Thus, the NPP dataset can be used as the proxy for statistical data allocation as it reflects crop growth to a certain extent. We collect the data made by Chen *et al*.^36^, which provides monthly NPP data for China’s terrestrial ecosystems from 1985 to 2015. We aggregate all data in 2015 and use it as a spatial proxy for the spatial allocation of the statistical data.

#### Marginal land identification

Marginal land can resolve the conflict between energy crop plantation, food security, and environmental pressure, which often indicates the area with low agricultural productivity and easy to degrade. However, marginal land does not have a clear definition and assessment procedure. Some studies identify marginal land based on land use types, believing that land not employed for a certain purpose (such as food production, forest plantation, grazing, etc.) may be categorized as marginal land^16,19^. Some studies identify the marginal land based on temporal changes in land use types. This approach categorizes abandoned cropland as marginal land as these areas have relatively fertile soils^37,38^. Even though there are still a variety of approaches and definitions^19,39–41^, this study identifies marginal land based on the aforementioned two principles since they nearly cover all the areas appropriate for energy crop plantation and their data processing procedure is straightforward.

1. identify the first type of marginal land based on land use types.
  First, we select the grids according to the land use types, which include shrub land, sparse land, high, medium, and low coverage grassland, intertidal zone, beach land, alkaline land, and bare land. Second, the grid cells in the natural reserve, slopes larger than 25 degrees, and the pasture area should be excluded. The pasture areas include the medium and high grassland in Xinjiang, Qinghai, Inner Mongolia, Ningxia, and Tibet. The final grids can be regarded as marginal land.
2. identify the second type of marginal land based on time series changes in agricultural land.

Abandoned cropland represents the areas that were agricultural land in the past but are not agricultural land at present. These areas have relatively good natural conditions and could be suitable for energy crop plantation. This study collects land use maps for 1980 and 2018 and assigns the value of arable land as one and the other areas as zero (as shown in Eq. (1)). Then, we calculate the abandoned cropland based on Eq. (2). In the end, according to the principle of biodiversity protection, the areas that overlap with natural reserves are excluded.

1 $${{\rm{LandUse}}}_{i,l,t}=\left\{\begin{array}{c}1,if\;grid\;i\;belongs\;to\;land\;use\;type\;l\\ 0,others\end{array}\right.$$

2 $${{\rm{ML}}}_{i}=\left(1-{{\rm{LandUse}}}_{i,l,t=2018}\right)-\left(1-{{\rm{LandUse}}}_{i,l,t=1980}\right),{\rm{l}}\in \left\{{\rm{cropland}}\right\}$$

Where, i represents the number of the grid cell. l represents the land use types. t represents the year. LandUse_*i, l, t*_ represents whether the grid cell i belongs to land use type l. ML_*i*_ represents whether the grid cell i is abandoned cropland.

### Agricultural residues resource potential assessment

The assessment of agricultural residues combines the statistical accounting method with the spatial allocation method. First, we collect the provincial data on agricultural products from China Statistic Yearbook in 2018, which includes 9 types of products, namely rice, maize, wheat, other grain, cotton, canola, peanuts, soybeans, and potatoes. Then, we collect the ratio of product to residues (RPR) and collectible ratio (CR) from the Regional Crop Straw Treatment and Utilization Technology Guideline published by the China Agricultural Ministry (http://www.reea.agri.cn/sttzgg/201702/t20170224_5494334.htm). The product to residues represents the ratio of economic products and straw products, the value of which is obtained based on the field investigation. In the collection process, part of the leaves and branches will be left in the field and the transportation process will also cause some losses. Thus, the final collectible biomass resource should also be multiplied by the collectible ratio (as shown in Eq. (3)).

3 $${A}_{k,p}={Y}_{k,p}\ast {\lambda }_{k,p}\ast {\eta }_{k,p}$$

Where, k denotes the type of agricultural product; p denotes the province; *A*_*k, p*_ (*tonne*) represents the total amount of agricultural residues sourced from feedstock k in province p; Y_*k,p*_ (*tonne*) represents the production of agricultural product k in province p; *λ*_*k,p*_ (%) and *η*_*k,p*_ (%) represent the RPR and CR of crop k in province p (Supplementary Tables 1, 2).

Based on the above provincial statistical results of agricultural residues, we combine the NPP data with land use maps for spatial allocation. First, land use maps are used as a mask to extract NPP data so that rice residues are distributed on the paddy land and other agricultural residues are distributed on the dry land. Then, we divide the NPP value of each grid cell by the total amount of provincial NPP data for calculating the spatial proxy to allocate the provincial amount of agricultural residues.

4 $$Pro{v}_{i,p}=\left\{\begin{array}{c}1,if\;grid\;i\;\in \;province\;p\\ 0,others\end{array}\right.$$

5 $$Agr{i}_{i,k}={A}_{k,p}\ast \frac{np{p}_{i}\ast landUs{e}_{i,l}\ast Pro{v}_{i,p}}{{\sum }_{p}np{p}_{i}\ast landUs{e}_{i,l}\ast Pro{v}_{i,p}}$$

Where, *Prov*_*i, p*_ represents whether this grid cell i belongs to province p; *Agri*_*i,k*_ (*tonne*/*km*^2^) represents the production of agricultural resides k in grid cell i; *npp*_*i*_ (*gC*/(*m*^2^*a)) represents the net primary value of grid cell i; *A*_*k,p*_ and *landUse*_*i, l*_ are the same as above.

### Forestry residues resource potential assessment

Forest residues in this study consist of three sources^14,42^, which are the residues derived from wood exploitation and process (including commercial wood, fuel wood, and logs), bamboo exploitation and process, and forest logging (including fuel forest, timber forest, protection forest, economic forest, special forest, shrub forest, and sparse forest).

For the residues derived from wood exploitation and process, we collect the production of commercial wood (not including firewood), firewood, and logs according to China Forestry and Grassland Statistical Yearbook in 2018^43^. Commercial wood refers to the trees or stands that can be sold as commodities after harvesting and timbering. They are sourced from natural forests or artificial forests. We use the outturn rate of merchantable timber to calculate the forestry residues derived from commercial wood (Eq. (6)). Even though firewood also belongs to commercial wood based on the definition, we calculate it separately as its production is specially used for energy production (Eq. (7)). Logs are pieces of wood that are cut to certain lengths. These woods will be further processed into wood products, such as building materials, furniture, and so on. This process will generate some waste, which can be used as forestry residues (Eq. (8)).

6 $${F}_{k=1,p}={{\rm{V}}}_{{\rm{mer}},{\rm{p}}}\ast {\rm{\rho }}\ast \frac{1-{{\rm{r}}}_{{\rm{mer}}}}{{{\rm{r}}}_{{\rm{mer}}}},{\rm{k}}\in \left\{{\rm{commercial}}\;{\rm{wood}}\right\}$$

7 $${F}_{k=2,p}={{\rm{V}}}_{{\rm{fire}},{\rm{p}}}\ast {\rm{\rho }},{\rm{k}}\in \left\{{\rm{firewood}}\right\}$$

8 $${F}_{k=3,p}={{\rm{V}}}_{{\rm{\log }},{\rm{p}}}\ast {\rm{\rho }}\ast {{\rm{r}}}_{{\rm{woodprocess}}},{\rm{k}}\in \left\{{\rm{logs}}\right\}$$

Where, *F*_*k,p*_ (tonne) represents k type of forestry residues in province p; V_*mer,p*_ (m^3^), V_fire,p_ (*m*^3^) and V_log,p_ (*m*^3^) represent the volume of the merchantable timber, firewood, and logs in province p, respectively; r_mer_ represents the outturn rate of merchantable timber (77.17%); r_woodProcess_ represents the residue rate when logs are processed into wood products (60%); ρ represents the air-dry density of the timber (0.618 t/m^3^). Parameters are sourced from Wang *et al*.^14^.

In the process of bamboo harvesting, leaves and branches can be used as forestry residues. The total amount of these residues is calculated according to the proportional relationship between the weight of leaves and branches and the weight of bamboo timber (r_leaf_). Additionally, when bamboo is processed into bamboo products, such as bamboo mats and beds, the bamboo residues generated in the process can also be used as forestry residues, which can be calculated according to the utilization rate of bamboo wood (r_bambooProcess_). We collect bamboo production data from China Forestry and Grassland Statistical Yearbook^43^, and calculate these residues according to Eq. (9).

9 $${F}_{k=4,p}={N}_{bamboo,p}\ast {\rm{g}}\ast {{\rm{r}}}_{{\rm{leaf}}}+{N}_{bamboo,p}\ast {\rm{g}}\ast {{\rm{r}}}_{{\rm{bambooProcess}}},{\rm{k}}\in \left\{{\rm{bamboo}}\;{\rm{wood}}\right\}$$

Where, *N*_*bamboo,p*_ represents the number of bamboo in province p, g represents the weight of unit bamboo (0.015 t/unit); r_leaf_ represents the ratio of the weight of bamboo leaf to the weight of the bamboo truck (38.07%); r_bambooProcess_ is determined by the utilization rate of bamboo wood, which equals 62% (1–38% = 62%). The values of these parameters are collected from Wang *et al*.^14^.

For the residues derived from forest logging, this study considers 7 types of forest, including fuel forest, timber forest, protection forest, economic forest, special forest, shrub forest, and sparse forest. The State Forestry Administrative of China established system of forest resources inventory every five years since the 1970s, which reflects the forest resource for each province. For the first 5 types of forestry residues, we collect the forest area data in each province based on the result of the ninth forest inventory from 2014 to 2018 (https://forest.ckcest.cn/sd/si/zgslzy.html). The total areas of these forests are 2.41 million km^2^, among which protection forest and timber forest account for 54.1% and 30.0%. According to the literature review, we collect two types of parameters for each forest in each province, including wood production rate (*α*_*k,p*_) and collectible ratio (*β*_*k,p*_). The wood production rate represents the wood production per unit area and the collectible ratio represents the ratio between logging residues and wood production. As provinces vary in latitude and topography, these parameters are different for each province. By multiplying the area with these parameters, we can obtain the above 5 types of logging residues in each province.

10 $${F}_{k,p}=Are{a}_{k,p}\ast {\alpha }_{k,p}\ast {\beta }_{k,p},{\rm{k}}\in \{{\rm{fuel}}\;{\rm{forest}},{\rm{timber}}\;{\rm{forest}},{\rm{protection}}\;{\rm{forest}},\;{\rm{economic}}\;{\rm{forest}},{\rm{special}}\;{\rm{forest}}\}$$

Where, *Area*_*k, p*_(*km*^2^) represents the area of forest k in province p; wood production rate (*α*_*k,p*_) and collectible ratio (*β*_*k,p*_) for forest type k in province p can be found in Supplementary Table 3. These parameters are collected from Wang *et al*.^14^ and Yuan *et al*.^44^.

To obtain the spatial distribution of the above 9 types of forestry residues, we need to use a land use map and NPP data for spatial allocation. Forest land consists of 4 sub-classes, including woodland, shrub forest, sparse forest, and other forest lands. The above types of forestry residues should be allocated to woodland, and their spatial allocation process is similar to agricultural residues, as shown in Eq. (11). For the logging residue source from shrub forest and sparse forest, as the land use maps contain these two land use types, we can calculate their spatial distribution directly based on the map as shown in Eq. (12).

11 $$\begin{array}{l}Fore{s}_{i,k}={F}_{k,p}\ast \frac{np{p}_{i}\ast landUs{e}_{i,l}\ast Pro{v}_{i,p}}{{\sum }_{p}np{p}_{i}\ast landUs{e}_{i,l}\ast Pro{v}_{i,p}},{\rm{l}}\,\in \,{\rm{woodland}},\\ {\rm{k}}\,\in \left\{\begin{array}{c}{\rm{commercial}}\;{\rm{wood,\; firewood,\; logs,\; bamboo,\; fuel}}\;{\rm{forest,\; timber}}\;{\rm{forest,}}\\ {\rm{protect}}\;{\rm{forest,\; economic}}\;{\rm{forest,\; special}}\;{\rm{forest}}\end{array}\right\}\end{array}$$

12 $$\begin{array}{l}Fore{s}_{i,k}=LandUs{e}_{i,l}\ast {\alpha }_{k,p}\ast {\beta }_{k,p},l\in \left\{shrub\;land,sparse\;land\right\},\\ \quad \quad \quad \quad k\in \left\{shrub\;forest,sparse\;forest\right\}\end{array}$$

Where, *Fores*_*i, k*_ (*tonne*/*km*^2^) represents the production of the kth forestry residue in grid cell i. Other annotations are the same as above.

### The spatial distribution of energy crops on marginal lands

For assessing the potential and the spatial distribution of energy crops, we need to collect two sets of maps. One is the yield map, which depicts the crop yield in each grid based on the natural conditions, such as temperature, precipitation, soil types, etc. The other map is the suitability map, which illustrates whether or not this grid is suitable for growing energy crops, taking into account human factors, such as natural reserves, water and soil conservation, agricultural protection, etc. We collect the global yield maps of energy crops from Li *et al*.^45,46^, which predict the yields of 2 types of herbaceous crops (miscanthus and switchgrass) and 3 types of woody crops (poplar, willow, and eucalypts). The authors also provide a best crop type map, which selects the highest yield for each 0.5° × 0.5° grid cell. Thus, we downscale this map to 1 km resolution and then overlapped the map with the above two types of marginal land.

### The spatial distribution map of bioenergy from different feedstock types

In the previous sections, we assess the biomass resource potential derived from agricultural residues, forestry residues, and energy crops separately. To have a comprehensive understanding of bioenergy potential, we need to aggregate these 9 types of agricultural residues, 11 types of forestry residues, and the 5 types of energy crops. It is worth noting that, simply summing up these datasets could cause overestimation as these datasets may overlap in space. The first type of marginal land selected by the land use type takes the sparse land and shrub land into account. The second type of marginal land identified by the land use change can only ensure the selected grid cells are not cropland. With the premise of ensuring food safety, protecting main forest land and pasture, and preserving biodiversity, each grid cell should provide bioenergy as much as possible. Therefore, we convert biomass production to energy content and select a feedstock type that can provide the maximum bioenergy amount for each grid cell. We assume the heat value of agricultural residues, forestry residues, and energy crops are 14.7, 17.3, and 16.3 GJ/t^47,48^, respectively.

In addition, as the achievable bioenergy potential should consider many realistic situations, we set five scenarios for the data aggregation of different feedstock types as shown in Table 1. The first scenario represents the maximum potential of bioenergy, as we consider all types of agricultural residues, forestry residues, and energy crops on all the marginal land. In the second scenario, energy crops will be grown on the first type of marginal land, which has a large total area but the productions in some grid cells are low. In the third scenario, energy crops will be grown on abandoned cropland, which has relatively good natural and human conditions as these areas were used to grow food in the past. The fourth scenario only includes agricultural and forestry residues as these two types of biomass resources are currently available. The last scenario is more in line with the biomass resources potential that is now accessible. In actuality, only a portion of residues could be used for energy production. The other residues would be utilized as organic soil fertilizer, raw materials for edible mushroom production, feed for herbivores, etc. Thus, we assume only 11% of agricultural and forestry residues could be used to produce bioenergy^49^. The total amount of bioenergy potential decrease gradually and get closer to reality from Scenario 1 to Scenario 5.

**Table 1:** Scenario setting for bioenergy potential.

Scenario settings	Agricultural residues	Forestry residues	Energy crops
S1	Yes	Yes	Yes, allowed to be planted on all available marginal land
S2	Yes	Yes	Yes, allowed to be planted on the first type of marginal land
S3	Yes	Yes	Yes, allowed to be planted on the second type of marginal land (abandoned cropland)
S4	Yes	Yes	No
S5	Yes, 11% for energy production	Yes, 11% for energy production	No

## Data Records

**Fig. 2:**
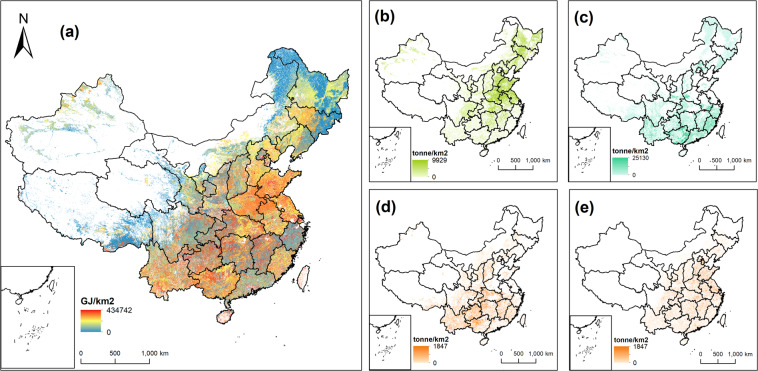
Biomass resource potential in China. (**a**) shows the maximum bioenergy potential in China, which includes agricultural residues, forestry residues, and energy crops on all marginal land. (**b**–**e**) show the yield of agricultural residues distributed on the cropland, forestry residues distributed on the forest land, and energy crops distributed on two different types of marginal land.

We provide three different format datasets for multidiscipline researchers. The first format of the dataset is the biomass resource potential maps stored in GeoTIFF files (.tif) with the WGS84 projection at approximately 1 km (0.01°) resolution (part of the maps are as shown in Fig. 2). It includes 10 agricultural residues maps (9 types of agricultural residues and one aggregated map), 12 forestry residues maps (11 types of forestry residues and one aggregated map), 12 energy crops maps (5 types of energy crops and the best crop type planted on two types of marginal land), and 5 bioenergy potential maps under different levels of development. The second format of the dataset is two NetCDF files. One file stores all the breakdown data for the biomass resources, including two types of marginal land and the above-mentioned maps of agricultural residue, forestry residue, and energy crop. The other file stores 5 matrices of 4000 * 7000 grid cells that records the bioenergy potential maps under 5 different scenarios. The third format of the dataset is an Excel file, which is the zonal summary of the above high spatial resolution maps at the provincial level. This dataset could be useful for many sub-national integrated assessment models. These files are all accessible via figshare^50^. The nomenclatures and additional information are listed in Table 2.

**Table 2:** Nomenclature of data files and detailed explanations.

Nomenclatures	Unit	Details
agri_*.tif (e.g. agri_rice.tif)	tonne/km^2^	9 maps for the residues of rice, maize, canola, wheat, peanuts, other grain, soybeans, cotton, and potatoes. 1 map summarizes all the agricultural residues named “agri_all.tif”.
fores_* _*.tif (e.g. fores_woodprocess_merchant.tif)	tonne/km^2^	11 maps for the residues of wood exploitation and process, bamboo exploitation and process, and forest logging. 1 map summarizes all the forestry residues named “forest_all.tif”.
eCrop_*_margin.tif (e.g. eCrop_miscanthus_margin.tif)	tonne/km^2^	5 maps for different types of energy crops planted on the first type of marginal land, and 1 map for the best crop type planted on the marginal land named “eCrops_best_margin.tif”
eCrop_*_abandon.tif (e.g. eCrop_miscanthus_abandon.tif)	tonne/km^2^	5 maps for different types of energy crops planted on the abandoned cropland, and 1 map for the best crop type planted on the abandoned cropland named “eCrops_best_abandon.tif”
bioenergy_*_*.tif (e.g. bioenergy_s1_AFE_max.tif)	GJ/km^2^	5 maps for bioenergy potential under the scenario 1 to scenario 5.
BioRes_breakdown.nc		36 variables, including 2 variables for marginal land (unit: km^2^); 10 variables for agricultural residues (tonne); 12 variables for forestry residues (tonne); 6 variables for energy crops on marginal land (tonne); 6 variables for energy crops on abandoned cropland (tonne). The annotations of these variables are the same as GeoTIFF files.
BioEnergy_scenario.nc		5 variables, including s1_AFE_max (GJ), s2_AFE_margin (GJ), s3_AFE_abandon (GJ), s4_AF_max (GJ), s5_AF_min (GJ)
provin_stat_map.xlsx		This file includes 5 sheets, which are the provincial statistics obtained by aggregating the above maps. Sheet 1 named “margin_land” provides the provincial results for 2 types of marginal land areas; Sheet 2 named “agri_res” provides the provincial results for 9 types of agricultural residues and their total amount; Sheet 3 named “fores_res” provides the provincial results for 11 types of forestry residues and their total amount; Sheet 4 named “eCrops” provides the provincial results for 5 types of energy crops and the best energy crops; Sheet 5 named “bioScen” provides provincial results of bioenergy potential under five different scenarios.

## Technical Validation

### Statistical analysis

**Fig. 3:**
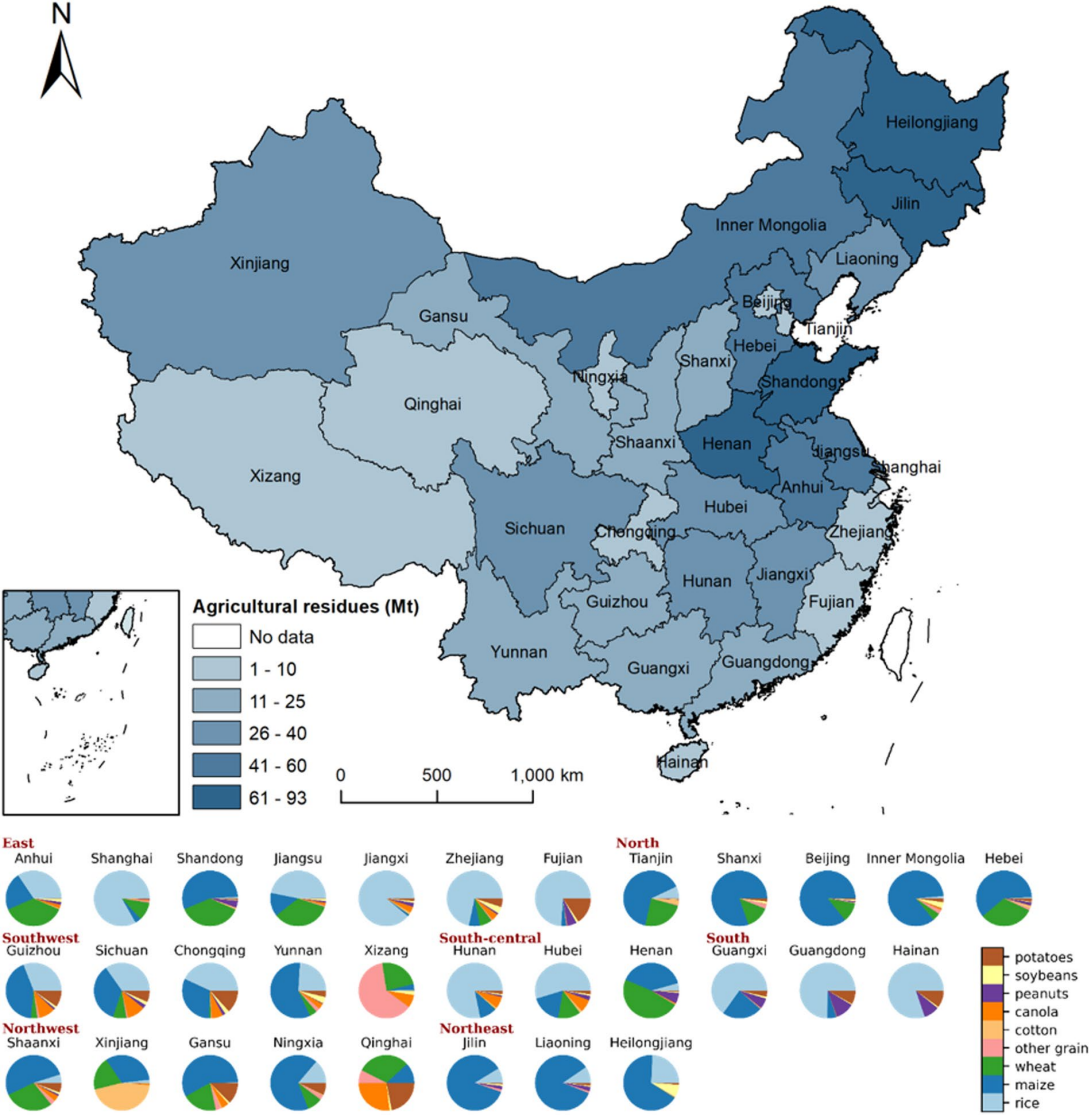
The biomass resource potential of agricultural residues in each province in China. Each province in China is colored according to the production of agricultural residues. Each color in the pie chart represents the type of agricultural residues.

Based on the high spatial resolution datasets of 9 types of agricultural residues, we can calculate the total agricultural residues amount for each province and understand the resource composition, as shown in Fig. 3. According to our results, Heilongjiang, Henan, and Shandong provinces have the largest agricultural residues, with 93, 86, and 75 million tonnes (Mt), respectively. The pie chart reveals that the resource composition of different regions or provinces is distinct. For North and Northeast China, the primary source of agricultural residues is maize straw. While in South-central, South, and East China, rice straw is the predominant agricultural residue. Some provinces have their distinct resource composition. For example, as Xinjiang province produces a considerable amount of cotton, the agricultural residues derived from cotton contribute more to the total. And for some provinces with high elevation, like Qinghai and Tibet, the residues derived from canola and other grain have a larger proportion.

**Fig. 4:**
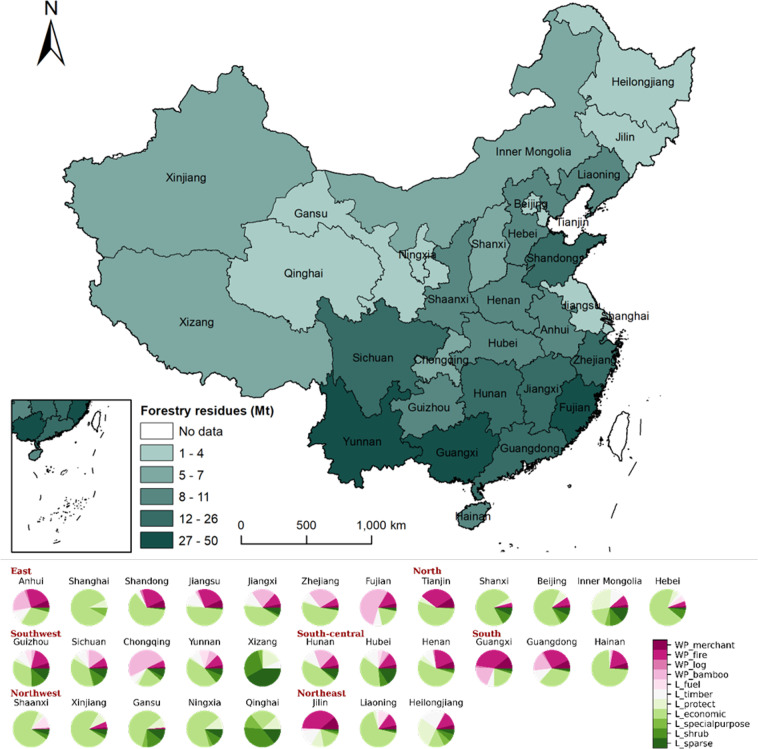
The biomass resource potential of forestry residues in each province in China. Each province in China is colored according to the production of forestry residues. Each color in the pie chart represents the type of forestry residues.

For forest residues, Guangxi, Yunnan, and Fujian provinces have the greatest potential, with 50, 33, and 26 Mt, respectively. The forestry residues of Guangxi province are mainly contributed by the logging residues of fuel forest and economic forest, as well as the exploitation and processing residues of bamboo. The residues of Yunnan province are contributed by the economic forest logging. And the bamboo forest logging residues are the predominant forestry residues for Fujian province. The pie chart in Fig. 4 reveals that the forest residues in Northwest China are mainly contributed by forest logging, while the provinces in East, South-central, and South China could explore the potential of forestry residues from wood exploitation and process. Some provinces have unique resource compositions, for example, Fujian and Chongqing provinces are rich in forestry residues from bamboo exploitation and process, while Tianjin, Jilin, and Guangxi provinces have a larger amount of fuel wood logging residues.

**Fig. 5:**
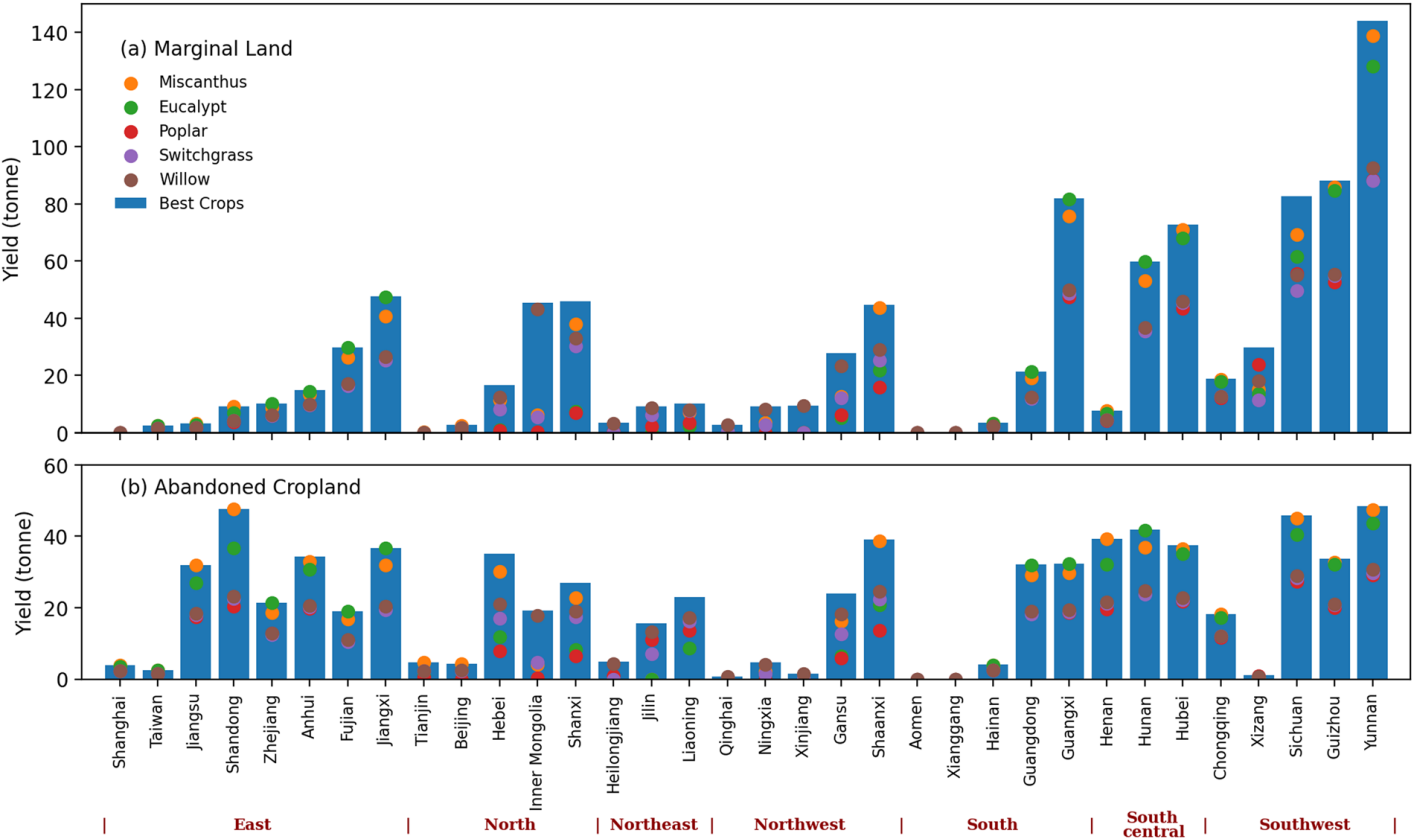
The biomass resource potential of energy crops on marginal land in each province in China. (**a,b**) represent the yield of energy crops that are planted on the first and second types of marginal land. The scatters represent the provincial yield for growing a particular type of energy crop on marginal land. The blue bar represents the provincial yield for growing the best crop type on marginal land.

In terms of energy crops, Southwest China is more suitable for energy crops plantation. According to our results, even though Xinjiang, Tibet, and Qinghai provinces have larger areas of the first type of marginal land with 296,671, 268,949, and 184,379 km^2^, respectively, Yunnan, Guizhou, and Sichuan provinces in Southwest China have larger biomass resource potential of energy crops, which are 144, 88, and 82 Mt if each grid cell is planted with the best crop type. In the second type of marginal land (abandoned cropland), Inner Mongolia, Yunnan, and Sichuan provinces have the largest areas of marginal land, with 42,117, 36,331, and 32,711 km^2^, respectively. However, as the yield of energy crops in each grid cell in Inner Mongolia is low, the total production of energy crops in Inner Mongolia can only reach 19.3 Mt. The total production of energy crops in Yunnan, Shandong, and Sichuan provinces can reach 48, 47, and 46 Mt. The scatters in Fig. 5 also shows the total production of each province if we only grow a single type of energy crops on the marginal land. In South, East, South-central, and Southwest China, the provinces can obtain higher production if they choose to plant miscanthus or eucalypt on the marginal land. But in North, Northwest, and Northeast China, planting willow or miscanthus would be a better option.

### Comparison with the existing studies

**Fig. 6:**
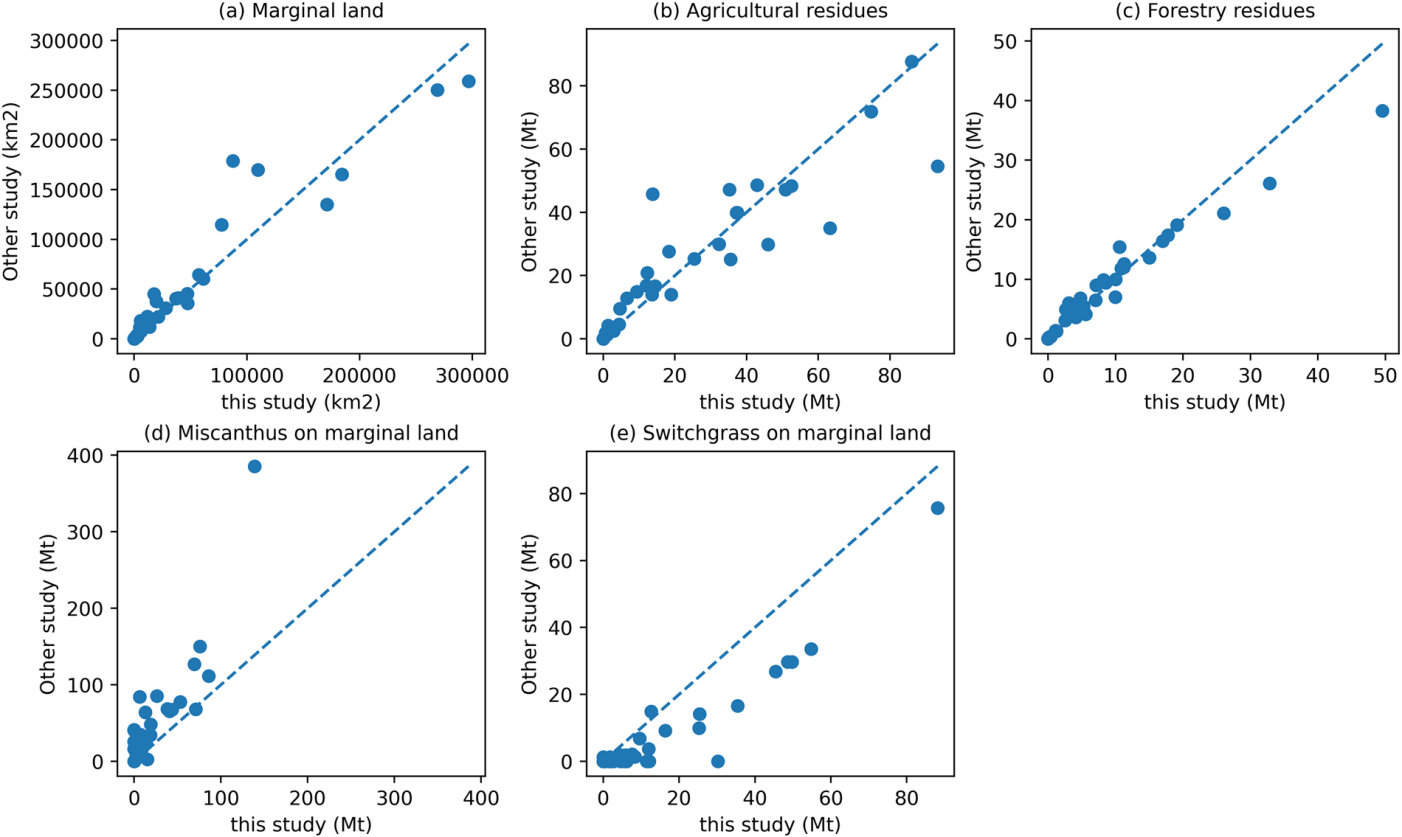
The comparison of the provincial data with the existing studies. The horizontal axis represents the provincial data of this study, and the vertical axis represents the data from the existing study. The data for (**a**), (**d**), and (**e**) are derived from Zhang *et al*.^16^. The data for (**b**) and (**c**) are derived from Bi *et al*.^9^ and Wang *et al*.^14^.

Due to the absence of a publicly available biomass resource dataset, we could not prove the accuracy of our dataset directly by comparing it with the same product. But we can prove the reliability of our dataset by aggregating our dataset at the provincial and national levels and then comparing them with the existing studies. The comparisons of the national results are shown in Table 3. For marginal land, our study indicates that 60.98 million hectares (Mha) of abandoned cropland and 167.53 Mha of marginal land could be utilized for energy crop plantation. Cai *et al*.^39^ use the fuzzy logic method to assess the land productivity and then estimate the land available for biofuel production under four scenarios. Their results show that China’s marginal land might range from 52 to 213 Mha, which is comparable to ours. Zhang *et al*.^16^ and Xue *et al*.^19^ estimate marginal land based on land use maps and the Chinese Academy of Science’s land use classification system, which is similar to our assessment method of the first type of marginal land. Their findings suggest 171 to 185 Mha of marginal land may be suitable for energy plantation, which is slightly bigger than our estimate. We believe this is because our study excludes the grid cells that overlap with the natural reserves in accordance with the principle of biodiversity protection. We compare our provincial data with Zhang *et al*.^16^ as shown in Fig. 6a. The scatters are distributed near the 1:1 diagonal line, which proves the similarity of our data with the existing research at the sub-national level. For agricultural residues, Bi *et al*.^9^ and Xing *et al*.^22^ show that the potential of China’s agricultural residues in 2008 and 2015 are 842 and 790 Mt, respectively, which is similar to our assessment for 2018. We also compare the provincial data of agricultural residues with Bi *et al*.^9^ as shown in Fig. 6b. In terms of forestry residues, the total amount of forestry residues derived from Wang *et al*.^14^ and Xing *et al*.^22^ are 303 and 310 Mt in 2013 and 2015, respectively, which is very similar to our assessment that 335.28 Mt of forestry residues could be available in 2018. At the provincial level, our dataset shows great consistency with the provincial data provided by Wang *et al*.^14^ as shown in Fig. 6c. For the potential of energy crops, Xing *et al*.^22^ show the potential of miscanthus on the marginal land range from 320 to 1940 Mt. Zhang *et al*.^16^ evaluate the potential of miscanthus, switchgrass, and Jatropha on the marginal land with 1761.1, 29.9, and 9.7 Mt, respectively. We compare our provincial data that only plant miscanthus or switchgrass on the first type of marginal land with the provincial results of Zhang *et al*.^16^, as shown in Fig. 6d,e. The scatters in Fig. 6d are slightly higher than the 1:1 diagonal line, which means our estimation of the miscanthus is lower than Zhang *et al*.^16^. While the estimation of switchgrass shows the opposite situation as the scatters are lower than the 1:1 diagonal line. We believe these variations are acceptable considering the differences between the studies in methodologies and these scatters are very close to the 1:1 diagonal line. In terms of national bioenergy potential, the results of this study from scenario 1 to scenario 5 are 1.9, 17.9, 29.3, 33.0, and 41.5 EJ, respectively. As Xing *et al*.^22^ use a constant heat value of 19 GJ/tonne to convert the production to energy content and do not exclude high-coverage grassland for grazing, their results are slightly higher than ours. Generally, by comparing our dataset with the existing studies, this dataset is credible no matter at the national or provincial level.

**Table 3:** The comparison of the national results with the existing studies.

	Results of the current study	Results of the relevant study
Marginal land	60.98~167.53 Mha	52~213 Mha^39^
		185 Mha^16^
		171.64 Mha^19^
Agricultural residues amount	849.56 Mt	842.19 Mt^9^
		790.0 Mt^22^
Forestry residues amount	335.28 Mt	302.83 Mt^14^
		310.0 Mt^22^
Energy crops amount	736.00~956.57 Mt	320~1940 Mt^22^
		2055 Mt^16^
Bioenergy potential	1.9~41.21 EJ	58 EJ^22^

### Comparison of the statistics and the maps

**Fig. 7:**
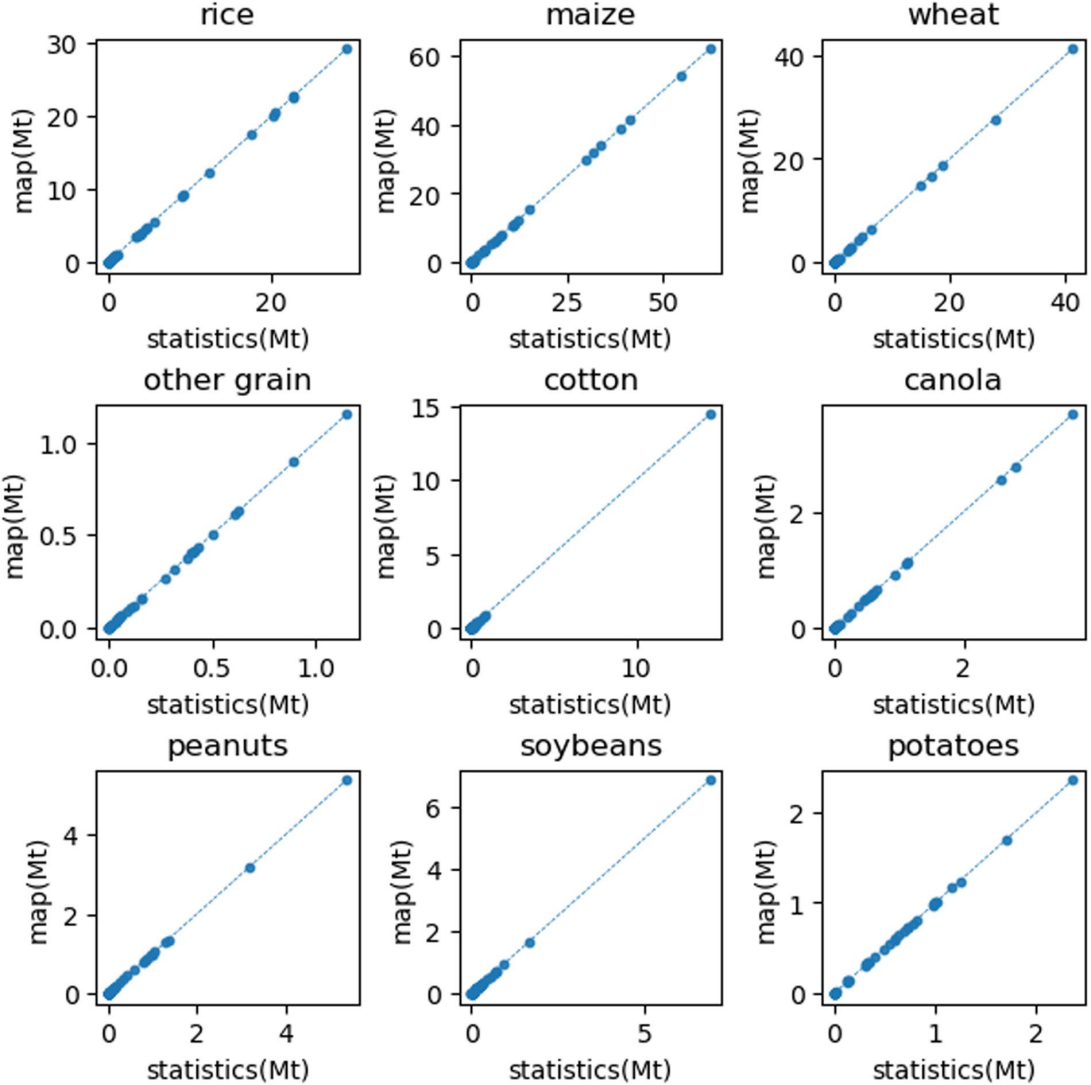
The comparison of the provincial agricultural residues statistics with the aggregated agricultural residues map data. The dotted line is a 1:1 diagonal line.

**Fig. 8:**
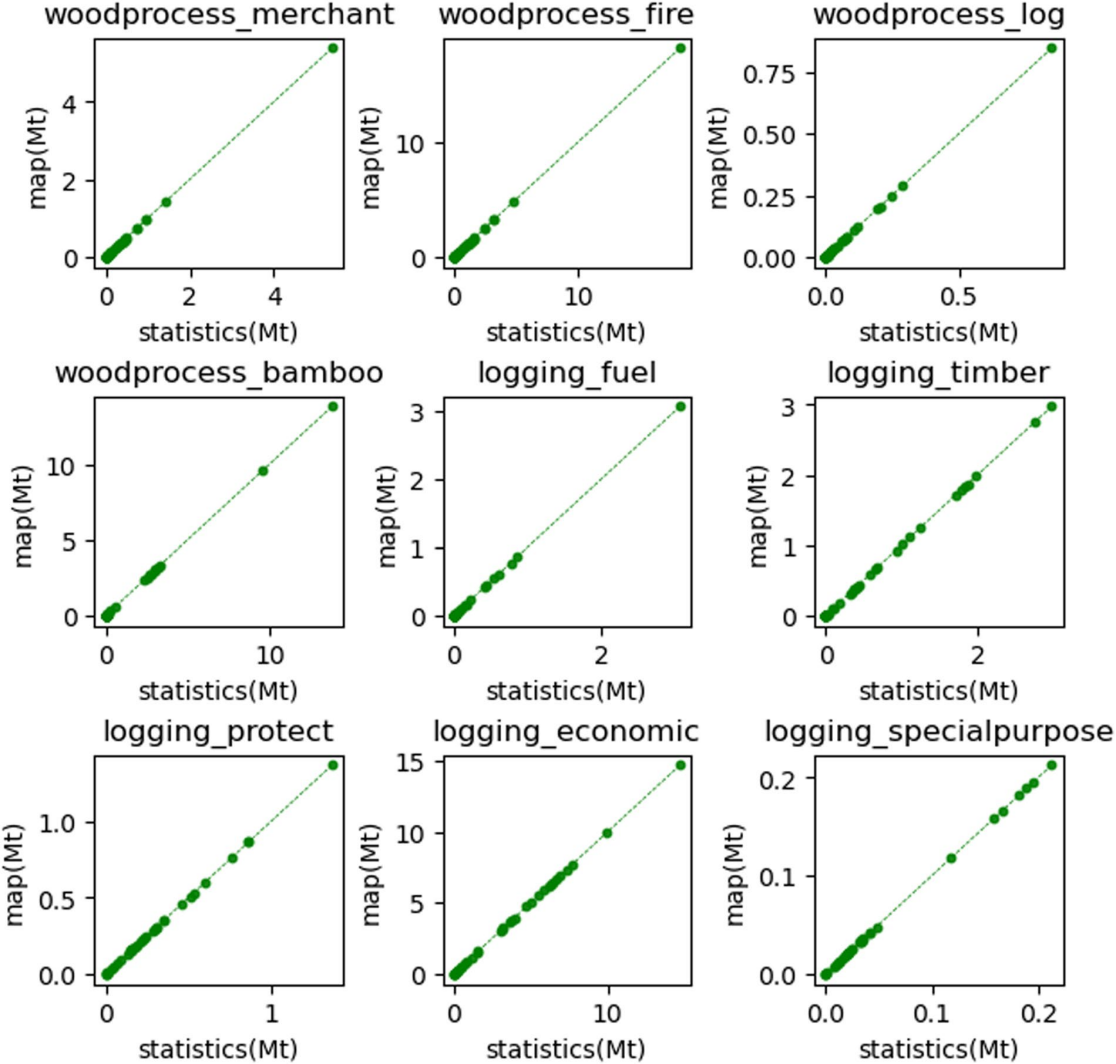
The comparison of the provincial forestry residues statistics with the aggregated forestry residues map results. The dotted line is a 1:1 diagonal line.

In addition, as this study combines the statistical accounting method with the GIS-based method for high spatial resolution assessment of biomass resources, to prove the consistency between the provincial statistics and the spatial allocation results, we aggregate high resolution maps at the provincial level and use scatters plots to compare these two results. Figure 7 shows the comparison results of 9 types of agricultural residues. As the logging residues of shrub land and sparse land are directly calculated based on the land use map, we only need to compare the remaining 9 types of forestry residues as shown in Fig. 8. These scatters are all distributed along the 1:1 diagonal line, which proves the consistency of our spatial allocation process.

In the end, there are three sources of uncertainties for our dataset. First, the uncertainty may derive from the conversion parameters. The parameters, such as the ratio of production to residues, collectible ratio, wood outturn rate, wood density, and the wood recycle rate, may result in deviation from reality. Even though this study collects the parameters that consider the heterogeneity of feedstock types and provinces, they could be further modified and improved in the future. Second, the uncertainty may derive from the statistics. The statistical data of agricultural products and forest areas are only available at the provincial level, which may introduce some bias in the process of data collection and compilation. Additionally, the forest investigation is conducted every five years. The ninth forest inventory data happened from 2014 to 2018. The volume of forest resources amount may change during these five years. Therefore, the assessment results of forestry residues may deviate from the actual results in 2018. Third, the uncertainty may derive from spatial data. Limited by the data availability, we could only obtain the NPP data in 2015, which could introduce a time bias. Besides, the land use map in 1980 could be less accurate than the land use map in 2018 due to the difference between the remote sensing satellites and the availability of supporting information, which could bring uncertainties for the identification of the abandoned cropland. Additionally, as the global energy crop map has a relatively coarse geographic resolution, the predicted yield of each grid may deviate from the actual yield of the land.

The uncertainties of our dataset could be improved from the following aspects. First, more studies or investigations can be conducted to improve the reliability of the conversion parameters. The construction of conversion parameters for different crop types and regions will serve as a crucial foundation for the assessment of biomass resource potential. Second, the development of county-level statistics data and more frequent forest investigation could also help to reduce uncertainties. Third, conducting energy crop plantations in more areas can provide more samples and basic information for the prediction of energy crops, hence improving the precision and geographic resolution of the energy crop yield map. In addition, producing more land use maps that can illustrate the spatial distribution of various agricultural products (such as the rice map, the cotton map, etc.) and the spatial distribution of forest use and management (such as the spatial distribution of economic forest map, fuel wood forest map, etc.), could also improve the accuracy of the high spatial resolution assessment of biomass resources.

## Usage Notes

This study provides a comprehensive dataset for almost all lignocellulosic biomass resources in China, which includes 9 types of agricultural residues, 11 types of forestry residues, and 5 types of energy crops on two different types of marginal land. As we provide spatial distribution maps for each type of biomass resource, users can aggregate these maps according to their needs. Besides, these maps can also help users understand which types of biomass resources contribute the most to the total amount in each region. In addition, to help users understand bioenergy potential in China at different levels of development, we aggregate the above maps based on five different scenarios. These maps can serve as important inputs for many Integrated Assessment Models and biomass industry chain optimization models. In the end, for different discipline researchers, we also provide three types of data formats, including NetCDF, GeoTIFF, and Excel. However, in our study, the dataset highly relies on national statistics, and the dataset production process of the agricultural residues and part of forestry residues is a downscaling exercise of provincial statistics. Thus, any error of the statistics will pass on to this dataset. We may expect that more studies on biomass resource assessment can make their dataset publicly available, which may be produced with different assessment framework, crop models or other data sources. It can help to improve the accuracy of the biomass resource dataset.

## Supplementary information

Supplementary Materials

## Data Availability

The code used for calculating agricultural, forestry residues, and energy crops is written in Python and available from https://github.com/Rui-W-A/biomass-resource-China.
