# Supplementary material for: A high spatial resolution dataset of China’s biomass resource potential
Source: Sci Data. 2023 Jun 15;10:384. doi: 10.1038/s41597-023-02227-7 (PMC10272228; doi:10.1038/s41597-023-02227-7)
Supplement: Supplementary file 1 — Supplementary Materials [file 41597_2023_2227_MOESM1_ESM.docx]

**A high spatial resolution dataset of China’s biomass resource potential**

**Authors**

Rui Wang^1^, Wenjia Cai^1,*^, Le Yu^1^, Wei Li^1^, Lei Zhu^1^, Bowen Cao^1^, Jin Li^2^, Jianxiang Shen^1^, Shihui Zhang^1^, Yaoyu Nie^3,4^, Can Wang^2^

Affiliations

1. Ministry of Education Ecological Field Station for East Asian Migratory Birds, Department of Earth System Science, Tsinghua University, Beijing 100084, China

2. State Key Joint Laboratory of Environment Simulation and Pollution Control (SKLESPC), School of Environment, Tsinghua University, Beijing 100084, China

3. PBC School of Finance, Tsinghua University, Beijing 100084, China

4. Beijing E-Hualu Information Technology Co., Ltd., Beijing 100043, China

corresponding author(s): Wenjia Cai, wcai@tsinghua.edu.cn

## Table S1. Parameter of the ratio of product to residues for the calculation of agricultural residues.

| OBJECTID | PingYing | rice | maize | wheat | other grain | cotton | canola | peanuts | soybeans | potatoes |
| --- | --- | --- | --- | --- | --- | --- | --- | --- | --- | --- |
| 1 | Anhui | 1.28 | 2.05 | 1.38 | 1.06 | 3.32 | 2.05 | 1.5 | 1.68 | 1.16 |
| 3 | Beijing | 0.93 | 1.73 | 1.34 | 0.85 | 3.99 | 2.02 | 1.22 | 1.57 | 1 |
| 4 | Chongqing | 1 | 1.29 | 1.31 | 0.97 | 3.65 | 2 | 1.42 | 1.05 | 0.6 |
| 5 | Fujian | 1.06 | 1.32 | 1.38 | 1.27 | 3.65 | 2.02 | 1.65 | 1.08 | 1.41 |
| 6 | Gansu | 1.06 | 1.52 | 1.23 | 1.23 | 3.67 | 2.02 | 1.42 | 1.07 | 1.22 |
| 7 | Guangdong | 1.06 | 1.32 | 1.38 | 1.27 | 3.65 | 2.02 | 1.65 | 1.08 | 1.41 |
| 8 | Guangxi | 1.06 | 1.32 | 1.38 | 1.27 | 3.65 | 2.02 | 1.65 | 1.08 | 1.41 |
| 9 | Guizhou | 1 | 1.29 | 1.31 | 0.97 | 3.65 | 2 | 1.42 | 1.05 | 0.6 |
| 10 | Hainan | 1.06 | 1.32 | 1.38 | 1.27 | 3.65 | 2.02 | 1.65 | 1.08 | 1.41 |
| 11 | Hebei | 0.93 | 1.73 | 1.34 | 0.85 | 3.99 | 2.02 | 1.22 | 1.57 | 1 |
| 12 | Heilongjiang | 0.97 | 1.86 | 0.93 | 0.97 | 3.65 | 2.02 | 1.42 | 1.7 | 1.71 |
| 13 | Henan | 0.93 | 1.73 | 1.34 | 0.85 | 3.99 | 2.02 | 1.22 | 1.57 | 1 |
| 14 | Hubei | 1.28 | 2.05 | 1.38 | 1.06 | 3.32 | 2.05 | 1.5 | 1.68 | 1.16 |
| 15 | Hunan | 1.28 | 2.05 | 1.38 | 1.06 | 3.32 | 2.05 | 1.5 | 1.68 | 1.16 |
| 16 | Jiangsu | 1.28 | 2.05 | 1.38 | 1.06 | 3.32 | 2.05 | 1.5 | 1.68 | 1.16 |
| 17 | Jiangxi | 1.28 | 2.05 | 1.38 | 1.06 | 3.32 | 2.05 | 1.5 | 1.68 | 1.16 |
| 18 | Jilin | 0.97 | 1.86 | 0.93 | 0.97 | 3.65 | 2.02 | 1.42 | 1.7 | 1.71 |
| 19 | Liaoning | 0.97 | 1.86 | 0.93 | 0.97 | 3.65 | 2.02 | 1.42 | 1.7 | 1.71 |
| 20 | Neimenggu | 0.93 | 1.73 | 1.34 | 0.85 | 3.99 | 2.02 | 1.22 | 1.57 | 1 |
| 21 | Ningxia | 1.06 | 1.52 | 1.23 | 1.23 | 3.67 | 2.02 | 1.42 | 1.07 | 1.22 |
| 22 | Qinghai | 1.06 | 1.52 | 1.23 | 1.23 | 3.67 | 2.02 | 1.42 | 1.07 | 1.22 |
| 23 | Shaanxi | 1.06 | 1.52 | 1.23 | 1.23 | 3.67 | 2.02 | 1.42 | 1.07 | 1.22 |
| 24 | Shandong | 0.93 | 1.73 | 1.34 | 0.85 | 3.99 | 2.02 | 1.22 | 1.57 | 1 |
| 25 | Shanghai | 1.28 | 2.05 | 1.38 | 1.06 | 3.32 | 2.05 | 1.5 | 1.68 | 1.16 |
| 26 | Shanxi | 0.93 | 1.73 | 1.34 | 0.85 | 3.99 | 2.02 | 1.22 | 1.57 | 1 |
| 27 | Sichuan | 1 | 1.29 | 1.31 | 0.97 | 3.65 | 2 | 1.42 | 1.05 | 0.6 |
| 29 | Tianjin | 0.93 | 1.73 | 1.34 | 0.85 | 3.99 | 2.02 | 1.22 | 1.57 | 1 |
| 31 | Xinjiang | 1.06 | 1.52 | 1.23 | 1.23 | 3.67 | 2.02 | 1.42 | 1.07 | 1.22 |
| 32 | Xizang | 1 | 1.29 | 1.31 | 0.97 | 3.65 | 2 | 1.42 | 1.05 | 0.6 |
| 33 | Yunnan | 1 | 1.29 | 1.31 | 0.97 | 3.65 | 2 | 1.42 | 1.05 | 0.6 |
| 34 | Zhejiang | 1.28 | 2.05 | 1.38 | 1.06 | 3.32 | 2.05 | 1.5 | 1.68 | 1.16 |

Note: These parameters are collected from the Regional Crop Straw Treatment and Utilization Technology Guideline published by the China Agricultural Ministry. http://www.reea.agri.cn/sttzgg/201702/t20170224_5494334.htm.

## Table S2. Parameter of collectible ratio for the calculation of agricultural residues. (Parameters are collected from the Regional Crop Straw Treatment and Utilization Technology Guideline published by the China Agricultural Ministry)

| OBJECTID | PingYing | rice | maize | wheat | other grain | cotton | canola | peanuts | soybeans | potatoes |
| --- | --- | --- | --- | --- | --- | --- | --- | --- | --- | --- |
| 1 | Anhui | 0.83 | 0.9 | 0.83 | 0.85 | 0.86 | 0.64 | 0.83 | 0.56 | 0.73 |
| 3 | Beijing | 0.83 | 0.9 | 0.83 | 0.85 | 0.86 | 0.64 | 0.83 | 0.56 | 0.73 |
| 4 | Chongqing | 0.83 | 0.9 | 0.83 | 0.85 | 0.86 | 0.64 | 0.83 | 0.56 | 0.73 |
| 5 | Fujian | 0.83 | 0.9 | 0.83 | 0.85 | 0.86 | 0.64 | 0.83 | 0.56 | 0.73 |
| 6 | Gansu | 0.83 | 0.9 | 0.83 | 0.85 | 0.86 | 0.64 | 0.83 | 0.56 | 0.73 |
| 7 | Guangdong | 0.83 | 0.9 | 0.83 | 0.85 | 0.86 | 0.64 | 0.83 | 0.56 | 0.73 |
| 8 | Guangxi | 0.83 | 0.9 | 0.83 | 0.85 | 0.86 | 0.64 | 0.83 | 0.56 | 0.73 |
| 9 | Guizhou | 0.83 | 0.9 | 0.83 | 0.85 | 0.86 | 0.64 | 0.83 | 0.56 | 0.73 |
| 10 | Hainan | 0.83 | 0.9 | 0.83 | 0.85 | 0.86 | 0.64 | 0.83 | 0.56 | 0.73 |
| 11 | Hebei | 0.83 | 0.9 | 0.83 | 0.85 | 0.86 | 0.64 | 0.83 | 0.56 | 0.73 |
| 12 | Heilongjiang | 0.83 | 0.9 | 0.83 | 0.85 | 0.86 | 0.64 | 0.83 | 0.56 | 0.73 |
| 13 | Henan | 0.83 | 0.9 | 0.83 | 0.85 | 0.86 | 0.64 | 0.83 | 0.56 | 0.73 |
| 14 | Hubei | 0.83 | 0.9 | 0.83 | 0.85 | 0.86 | 0.64 | 0.83 | 0.56 | 0.73 |
| 15 | Hunan | 0.83 | 0.9 | 0.83 | 0.85 | 0.86 | 0.64 | 0.83 | 0.56 | 0.73 |
| 16 | Jiangsu | 0.83 | 0.9 | 0.83 | 0.85 | 0.86 | 0.64 | 0.83 | 0.56 | 0.73 |
| 17 | Jiangxi | 0.83 | 0.9 | 0.83 | 0.85 | 0.86 | 0.64 | 0.83 | 0.56 | 0.73 |
| 18 | Jilin | 0.83 | 0.9 | 0.83 | 0.85 | 0.86 | 0.64 | 0.83 | 0.56 | 0.73 |
| 19 | Liaoning | 0.83 | 0.9 | 0.83 | 0.85 | 0.86 | 0.64 | 0.83 | 0.56 | 0.73 |
| 20 | Neimenggu | 0.83 | 0.9 | 0.83 | 0.85 | 0.86 | 0.64 | 0.83 | 0.56 | 0.73 |
| 21 | Ningxia | 0.83 | 0.9 | 0.83 | 0.85 | 0.86 | 0.64 | 0.83 | 0.56 | 0.73 |
| 22 | Qinghai | 0.83 | 0.9 | 0.83 | 0.85 | 0.86 | 0.64 | 0.83 | 0.56 | 0.73 |
| 23 | Shaanxi | 0.83 | 0.9 | 0.83 | 0.85 | 0.86 | 0.64 | 0.83 | 0.56 | 0.73 |
| 24 | Shandong | 0.83 | 0.9 | 0.83 | 0.85 | 0.86 | 0.64 | 0.83 | 0.56 | 0.73 |
| 25 | Shanghai | 0.83 | 0.9 | 0.83 | 0.85 | 0.86 | 0.64 | 0.83 | 0.56 | 0.73 |
| 26 | Shanxi | 0.83 | 0.9 | 0.83 | 0.85 | 0.86 | 0.64 | 0.83 | 0.56 | 0.73 |
| 27 | Sichuan | 0.83 | 0.9 | 0.83 | 0.85 | 0.86 | 0.64 | 0.83 | 0.56 | 0.73 |
| 29 | Tianjin | 0.83 | 0.9 | 0.83 | 0.85 | 0.86 | 0.64 | 0.83 | 0.56 | 0.73 |
| 31 | Xinjiang | 0.83 | 0.9 | 0.83 | 0.85 | 0.86 | 0.64 | 0.83 | 0.56 | 0.73 |
| 32 | Xizang | 0.83 | 0.9 | 0.83 | 0.85 | 0.86 | 0.64 | 0.83 | 0.56 | 0.73 |
| 33 | Yunnan | 0.83 | 0.9 | 0.83 | 0.85 | 0.86 | 0.64 | 0.83 | 0.56 | 0.73 |
| 34 | Zhejiang | 0.83 | 0.9 | 0.83 | 0.85 | 0.86 | 0.64 | 0.83 | 0.56 | 0.73 |

Note: These parameters are collected from the Regional Crop Straw Treatment and Utilization Technology Guideline published by the China Agricultural Ministry. http://www.reea.agri.cn/sttzgg/201702/t20170224_5494334.htm.

## Table S3. Parameter for the calculation of intermediate cutting residues.

| OBJECTID | PingYing | area | Fuel_$\beta$ | Fuel_$\alpha$ | Timber_$\beta$ | timber_$\alpha$ | Protect_$\beta$ | protect_$\alpha$ | Economic_$\beta$ | economic_$\alpha$ | Special_$\beta$ | special_$\alpha$ | Street_$\beta$ | street_$\alpha$ | Shrub_$\beta$ | shrub_$\alpha$ | Spars_$\beta$ | sparse_$\alpha$ |
| --- | --- | --- | --- | --- | --- | --- | --- | --- | --- | --- | --- | --- | --- | --- | --- | --- | --- | --- |
| 1 | Anhui | plain and hill | 1 | 750 | 0.7 | 75 | 0.5 | 37.5 | 0.7 | 850 | 0.5 | 37.5 | 1 | 0.002 | 0.7 | 75 | 0.7 | 120 |
| 3 | Beijing | plain and hill | 1 | 750 | 0.7 | 75 | 0.5 | 37.5 | 0.7 | 850 | 0.5 | 37.5 | 1 | 0.002 | 0.7 | 75 | 0.7 | 120 |
| 4 | Chongqing | south mountain | 1 | 750 | 0.5 | 75 | 0.2 | 37.5 | 0.7 | 850 | 0.2 | 37.5 | 1 | 0.002 | 0.5 | 75 | 0.5 | 120 |
| 5 | Fujian | south mountain | 1 | 750 | 0.5 | 75 | 0.2 | 37.5 | 0.7 | 850 | 0.2 | 37.5 | 1 | 0.002 | 0.5 | 75 | 0.5 | 120 |
| 6 | Gansu | north mountain | 1 | 375 | 0.2 | 60 | 0.2 | 37.5 | 0.7 | 750 | 0.2 | 37.5 | 1 | 0.002 | 0.3 | 75 | 0.3 | 120 |
| 7 | Guangdong | south mountain | 1 | 750 | 0.5 | 75 | 0.2 | 37.5 | 0.7 | 850 | 0.2 | 37.5 | 1 | 0.002 | 0.5 | 75 | 0.5 | 120 |
| 8 | Guangxi | south mountain | 1 | 750 | 0.5 | 75 | 0.2 | 37.5 | 0.7 | 850 | 0.2 | 37.5 | 1 | 0.002 | 0.5 | 75 | 0.5 | 120 |
| 9 | Guizhou | south mountain | 1 | 750 | 0.5 | 75 | 0.2 | 37.5 | 0.7 | 850 | 0.2 | 37.5 | 1 | 0.002 | 0.5 | 75 | 0.5 | 120 |
| 10 | Hainan | south mountain | 1 | 750 | 0.5 | 75 | 0.2 | 37.5 | 0.7 | 850 | 0.2 | 37.5 | 1 | 0.002 | 0.5 | 75 | 0.5 | 120 |
| 11 | Hebei | plain and hill | 1 | 750 | 0.7 | 75 | 0.5 | 37.5 | 0.7 | 850 | 0.5 | 37.5 | 1 | 0.002 | 0.7 | 75 | 0.7 | 120 |
| 12 | Heilongjiang | north mountain | 1 | 375 | 0.2 | 60 | 0.2 | 37.5 | 0.7 | 750 | 0.2 | 37.5 | 1 | 0.002 | 0.3 | 75 | 0.3 | 120 |
| 13 | Henan | plain and hill | 1 | 750 | 0.7 | 75 | 0.5 | 37.5 | 0.7 | 850 | 0.5 | 37.5 | 1 | 0.002 | 0.7 | 75 | 0.7 | 120 |
| 14 | Hubei | south mountain | 1 | 750 | 0.5 | 75 | 0.2 | 37.5 | 0.7 | 850 | 0.2 | 37.5 | 1 | 0.002 | 0.5 | 75 | 0.5 | 120 |
| 15 | Hunan | south mountain | 1 | 750 | 0.5 | 75 | 0.2 | 37.5 | 0.7 | 850 | 0.2 | 37.5 | 1 | 0.002 | 0.5 | 75 | 0.5 | 120 |
| 16 | Jiangsu | plain and hill | 1 | 750 | 0.7 | 75 | 0.5 | 37.5 | 0.7 | 850 | 0.5 | 37.5 | 1 | 0.002 | 0.7 | 75 | 0.7 | 120 |
| 17 | Jiangxi | south mountain | 1 | 750 | 0.5 | 75 | 0.2 | 37.5 | 0.7 | 850 | 0.2 | 37.5 | 1 | 0.002 | 0.5 | 75 | 0.5 | 120 |
| 18 | Jilin | north mountain | 1 | 375 | 0.2 | 60 | 0.2 | 37.5 | 0.7 | 750 | 0.2 | 37.5 | 1 | 0.002 | 0.3 | 75 | 0.3 | 120 |
| 19 | Liaoning | north mountain | 1 | 375 | 0.2 | 60 | 0.2 | 37.5 | 0.7 | 750 | 0.2 | 37.5 | 1 | 0.002 | 0.3 | 75 | 0.3 | 120 |
| 20 | Neimenggu | north mountain | 1 | 375 | 0.2 | 60 | 0.2 | 37.5 | 0.7 | 750 | 0.2 | 37.5 | 1 | 0.002 | 0.3 | 75 | 0.3 | 120 |
| 21 | Ningxia | north mountain | 1 | 375 | 0.2 | 60 | 0.2 | 37.5 | 0.7 | 750 | 0.2 | 37.5 | 1 | 0.002 | 0.3 | 75 | 0.3 | 120 |
| 22 | Qinghai | north mountain | 1 | 375 | 0.2 | 60 | 0.2 | 37.5 | 0.7 | 750 | 0.2 | 37.5 | 1 | 0.002 | 0.3 | 75 | 0.3 | 120 |
| 23 | Shaanxi | north mountain | 1 | 375 | 0.2 | 60 | 0.2 | 37.5 | 0.7 | 750 | 0.2 | 37.5 | 1 | 0.002 | 0.3 | 75 | 0.3 | 120 |
| 24 | Shandong | plain and hill | 1 | 750 | 0.7 | 75 | 0.5 | 37.5 | 0.7 | 850 | 0.5 | 37.5 | 1 | 0.002 | 0.7 | 75 | 0.7 | 120 |
| 25 | Shanghai | plain and hill | 1 | 750 | 0.7 | 75 | 0.5 | 37.5 | 0.7 | 850 | 0.5 | 37.5 | 1 | 0.002 | 0.7 | 75 | 0.7 | 120 |
| 26 | Shanxi | north mountain | 1 | 375 | 0.2 | 60 | 0.2 | 37.5 | 0.7 | 750 | 0.2 | 37.5 | 1 | 0.002 | 0.3 | 75 | 0.3 | 120 |
| 27 | Sichuan | south mountain | 1 | 750 | 0.5 | 75 | 0.2 | 37.5 | 0.7 | 850 | 0.2 | 37.5 | 1 | 0.002 | 0.5 | 75 | 0.5 | 120 |
| 29 | Tianjin | plain and hill | 1 | 750 | 0.7 | 75 | 0.5 | 37.5 | 0.7 | 850 | 0.5 | 37.5 | 1 | 0.002 | 0.7 | 75 | 0.7 | 120 |
| 31 | Xinjiang | north mountain | 1 | 375 | 0.2 | 60 | 0.2 | 37.5 | 0.7 | 750 | 0.2 | 37.5 | 1 | 0.002 | 0.3 | 75 | 0.3 | 120 |
| 32 | Xizang | south mountain | 1 | 750 | 0.5 | 75 | 0.2 | 37.5 | 0.7 | 850 | 0.2 | 37.5 | 1 | 0.002 | 0.5 | 75 | 0.5 | 120 |
| 33 | Yunnan | south mountain | 1 | 750 | 0.5 | 75 | 0.2 | 37.5 | 0.7 | 850 | 0.2 | 37.5 | 1 | 0.002 | 0.5 | 75 | 0.5 | 120 |
| 34 | Zhejiang | south mountain | 1 | 750 | 0.5 | 75 | 0.2 | 37.5 | 0.7 | 850 | 0.2 | 37.5 | 1 | 0.002 | 0.5 | 75 | 0.5 | 120 |

Note: These parameters are sourced from Wang, H., Zuo, X., Wang, D. & Bi, yuyun. The estimation of forest residue resources in China. *Jounal Cent. South Univ. For. Technol.* **37**, 29–38 (2017).
